# Accounting for Dependence Induced by Weighted KNN Imputation in Paired Samples, Motivated by a Colorectal Cancer Study

**DOI:** 10.1371/journal.pone.0119876

**Published:** 2015-04-07

**Authors:** Anvar Suyundikov, John R. Stevens, Christopher Corcoran, Jennifer Herrick, Roger K. Wolff, Martha L. Slattery

**Affiliations:** 1 Department of Mathematics and Statistics, Utah State University, 3900 Old Main Hill, Logan, UT 84322-3900, U.S.A.; 2 Division of Epidemiology, Department of Internal Medicine, University of Utah School of Medicine, 383 Colorow Road, Salt Lake City, UT 84108, U.S.A.; National Taiwan University, TAIWAN

## Abstract

Missing data can arise in bioinformatics applications for a variety of reasons, and imputation methods are frequently applied to such data. We are motivated by a colorectal cancer study where miRNA expression was measured in paired tumor-normal samples of hundreds of patients, but data for many normal samples were missing due to lack of tissue availability. We compare the precision and power performance of several imputation methods, and draw attention to the statistical dependence induced by K-Nearest Neighbors (KNN) imputation. This imputation-induced dependence has not previously been addressed in the literature. We demonstrate how to account for this dependence, and show through simulation how the choice to ignore or account for this dependence affects both power and type I error rate control.

## Introduction

MicroRNAs (miRNAs) are small non-coding RNA molecules that regulate gene expression by targeting messenger RNAs. They were first discovered in 1993 during a study into development in the nematode Caenorhabditis elegans (C. elegans) regarding the protein gene lin-14 [1]. Lee et al. (1993) found that the abundance of protein lin-14 was regulated by a small RNA encoded by the lin-4 locus. This was transcribed into a 22-nucleotide RNA molecule that could repress the expression of the lin-14 messenger RNA (mRNA) by directly interacting with its 3’ untranslated region (UTR).

The scientific community is currently highly interested in the functional roles of miRNAs. The miRNA biogenesis that functions properly results in the normal rates of cellular growth, proliferation, differentiation, and cell death. But the reduction or deletion of miRNAs that is caused by defects at any stage of miRNA biogenesis leads to inappropriate expression of the miRNA-target oncoproteins that causes increasing proliferation, invasiveness or angiogenesis, or decreasing levels of apoptosis [2, 3].

The miRBase database, a searchable database of published miRNA sequences and annotation, had listed 2,588 unique mature human miRNAs for July 2014 (from http://www.mirbase.org). Since miRNAs can regulate more than one target, they may regulate up to more than 30% of all protein-coding genes in the human genome (from http://www.mirnarx.com). This makes miRNAs one of the largest regulators of gene expression.

The association between miRNAs and colorectal cancer (CRC) was reported for the first time in 2003, when the miR-143 and miR-145 genes were downregulated in CRC tumor tissues compared with normal tissues [4]. Since then, several studies have shown that miRNAs are extensively deregulated in CRC [5–7].

The miRNA data as most other expression data can be considered in the form of large matrices of expression levels of features (rows) in different subjects (columns). The data sets might have either some features missing in some samples, or all features missing in some samples. The former case often occurs due to insufficient resolution, image corruption, dust or scratches on the slide, and other various experimental and technical reasons, while the latter case may happen due to lack of collected tissue or limited funds. As an example of the latter case, we present the case study from research to determine the association of miRNAs with CRC in paired normal-tumor samples. As part of a preliminary analysis using the first available subjects, we wanted to compare miRNA expression profiles of normal and tumor samples from each of more than 400 subjects with 2006 miRNA on each sample. We also collected extensive information about demographic and lifestyle variables of these CRC patients. There are not many CRC studies that have collected such extensive data for such variables. However, in the final analysis using all available subjects, 10% to 50% of the subjects will have missing normal samples due to lack of tissue availability.

The immediate objective in this CRC case study is to understand the alternatives for imputation, along with their comparative strengths and weaknesses. Specifically, we wish to know for a given imputation method whether its application to missing miRNA data among normal samples will yield accurate predictions of their actual expression levels, and how such predictions are further affected by the percentage of subjects with missing values. We further wish to understand how these results affect statistical power to detect differentially expressed miRNA while controlling for Type I error.

With the proliferation of gene expression studies over the past decade, more attention has been paid to imputation methods for miRNA data. Conventional approaches often involve simply excluding miRNAs with missing values, replacing missing values with zeroes, or imputing using row or column averages. Such options ignore the correlation structure of the data and have limited power [8]. Moreover, they do not leverage potentially informative demographic or lifestyle variables. More sophisticated options use multiple imputation based on Markov Chain Monte Carlo (MCMC) and Expectation-Maximization (EM) algorithms, which allow the incorporation of additional covariates [9–11]

In this paper, we introduce and evaluate an imputation method that accounts for the dependence induced by weighted K-Nearest Neighbor (KNN) and considers the covariates, over the multiple imputation techniques using MCMC and EM with bootstrapping algorithms, as well as the case deletion technique using characteristics of this large CRC data set.

This paper is arranged in the following manner: first, we provide an overview of imputation assumptions and methods, as well as the RMSE method to assess the performance of various imputation techniques. Then we demonstrate the application of imputation techniques using simulation data sets. Finally, we conclude with a discussion of the important issues presented in the paper, such as the performance of the KNN imputation method while considering the dependence over the multiple imputation techniques.

## Methods

Before performing an imputation of missing data, it is necessary to know whether the missing data occurs randomly, as the result of unobserved factors, or is intended. We need to take into consideration two assumptions: missing at random (MAR) and missing completely at random (MCAR) [12]. The missing data are MAR when missing values are not randomly distributed across all observations but are randomly distributed within one or more subsamples of data. A variable (miRNA or *x*) can be considered MAR if the probability of observing *x* (conditional on observed variables) does not depend on *x*. The MCAR assumption is a special case of MAR, when the missing data values are a simple random sample of all data values. One can define the missing data as a missing not at random (MNAR) if neither MCAR nor MAR assumptions hold. In this case, missing data cannot be imputed based on the available data. Thus, imputation techniques can only be applied to the data which satisfy either MAR or MCAR assumptions. The characteristics of the CRC miRNA data satisfy MAR assumptions because the probability of subjects having missing normal samples does not depend on the miRNA expression values in those subjects.

We consider the following methods to estimate the miRNA expression levels for missing normal samples of patients:

### Multiple imputation

Multiple imputation (MI) was originally designed to handle missingness in public-use large data sets [12]. The application of the MI process has been extended to various big data sets including microarrays [13]. The method replaces each missing value with multiple substitute values, say *m*, that represent the probability distribution of the missing value. A completed dataset is created by each set of draws. So the *m* imputations for each missing value create *m* complete data sets. They are stored in an auxiliary matrix, multiply-imputed data sets with one row for missing value and *m* columns. The first row of this matrix corresponds to the first set of imputed values of the missing values, and so on. As the complete-data analyses are applied to each multiply-imputed dataset (treating imputed values as fully observed and independent), *m* different sets of the parameter estimates and their variance-covariance matrices are generated. To combine the inferences from them, [12] suggests to take an average of all results, except the standard error (SE) term. The SE is constructed by the within variance of each dataset as well as the variance between imputed items on each dataset. These two variances are added together and the square root of them determines the SE. The author recommends to use no more than 5 imputations and sometimes as small number as 2 or 3 to generate useful statistical inferences. We use *m* = 5 for MI techniques in our analysis. It is important to note that the complete-data analyses in MI treat the imputed data as though they had been fully observed. This approach does not consider any dependence of the imputed data on the actual fully observed data.

### MI using Markov chain Monte Carlo (MCMC)

Multiple imputed data sets can be generated by the MCMC method, which is applied to an arbitrary missing data pattern that assumes multivariate normality. MCMC has been used to explore posterior probability distributions to express unknown parameters in Bayesian inferences. Using this method, the entire joint posterior distribution of the unknown quantities is simulated and the parameter estimates based on the simulation are generated [14].

This process can be described in two steps. The first step is the imputation I-step which randomly draws values for missing values from the assumed distribution of missing values given observed values using the estimated mean vector and variance-covariance matrix, i.e. it draws values for $Y_{mis}^{\left(t+1\right)}$ from *p*(*Y*_*mis*_|*Y*_*obs*_, *θ* ^*t*^), where *Y*_*mis*_ and *Y*_*obs*_ are variables with missing values and observed values, respectively, and *θ* ^*t*^ is a parameter estimate at the *t* ^*th*^ iteration.

The posterior P-step randomly simulates the population mean vector and variance-covariance matrix from the complete sample estimates, i.e. it draws *θ* ^(*t*+1)^ from $p(\theta |Y_{obs},Y_{mis}^{\left(t+1\right)})$. These new estimates are then used in the I-step. This creates a Markov chain $\left(Y_{mis}^{\left(1\right)},\theta^{\left(1\right)}\right)$, $\left(Y_{mis}^{\left(2\right)},\theta^{\left(2\right)}\right)$, …, which converges in distribution to *p*(*Y*_*mis*_, *θ*|*Y*_*obs*_). Enough iterations are carried out to have reliable results for a multiply imputed dataset and to converge to its stationary distribution from which we can simulate an approximately random draw of the missing values [15].

### MI using Expectation-Maximization (EM) with bootstrapping algorithms

The EM algorithm is a very general iterative algorithm for maximum likelihood estimation of missing data [9]. One assumes a model for the data, maximizes the likelihood under the assumed model, obtains parameter estimates, and makes inferences based on the parameter estimates. The explicit form of parameter estimates does not usually exist for missing data. Here numerical methods like the Newton-Raphson algorithm are very complicated to use. Thus one can apply the EM algorithm which is an iterative method for maximizing the likelihood in missing data [10]. Compared to the Newton-Raphson algorithm, the EM algorithm is slower, but it increases the likelihood with each iteration and surely converges to a maximum for the distribution with one mode. The EM algorithm converges to a local maximum or a saddle point for the distribution with multiple modes.

The EM algorithm consists of two steps, the Expectation (E) and the Maximization (M) steps. The algorithm calculates the conditional expectation of missing values given non-missing values and current parameter estimates in the expectation step. In the maximization step the calculated expected values are used to maximize the likelihood of the complete data. These steps are iterated until the maximum likelihood of data converges. The EM algorithm may not have an explicit form. In this case, the maximization could be theoretically obtained using iterations in the maximization step.

The maximization step can be computationally expensive, which can make the EM algorithm unattractive. Fortunately, the EM with bootstrapping algorithm resolves this problem. It uses the conventional EM algorithm on multiple bootstrapped samples of the original missing data to draw values of the complete-data parameters. Then it draws imputed values from each set of bootstrapped parameters, replacing the missing values with these draws. The EM with bootstrapping algorithm can impute missing values in much less time than the EM algorithm itself [11].

### K-Nearest Neighbors (KNN): modified and accounting for dependence KNN in general

The conventional KNN method replaces missing values using *k*-most similar non-missing subjects’ values [16, 17]. It can impute both discrete attributes (using the most frequent value among the k-nearest neighbors) and continuous attributes (using the mean among the k-nearest neighbors).

[8] implemented the KNN method that weights the contribution of each nearest neighbor by its similarity to the subject with the missing value. In our CRC study, the weights of the nearest neighbors in the imputation of missing value are measured by the Euclidean distance metrics of demographic and lifestyle variables such that the nearer neighbors to the subject contribute more to its imputation than the more distant ones. Based on the weighting method of [8], we briefly outline our weight calculations here. Let *k* be the chosen number of nearest neighbors, *D*_*i*_1__ ≤ … ≤ *D*_*i*_*k*__ be the sorted distances of the *k* nearest neighbors from normal-missing subject *i*, and $D_{i}^{\left(max\right)}$ be the maximum distance (among all fully-observed subjects) from subject *i*. Then the weights *a*_*i*_1__, …, *a*_*i*_*k*__ among the *k* nearest neighbors for subject *i* are obtained as follows:

$$\begin{array}{ccc} w_{i_{l}} & = & 1-\frac{D_{i_{l}}}{D_{i}^{\left(max\right)}}\\ a_{i_{l}} & = & \frac{w_{i_{l}}}{\sum_{t=1}^{k}w_{i_{t}}} \end{array}$$ (1)

These weights are used by the weighted KNN method to impute missing expression values of a particular gene as in Eq (2).

Our proposed imputation method accounts for the dependence induced by weighted KNN and can use the additional covariates such as demographic, general health, genetic, and lifestyle variables, as well as other biologically related information. The proposed imputation method takes advantage of the conventional KNN [16, 17] and further developed weighted KNN [8] imputation methods’ robustness to missing data, non-parametric approach, and speed in estimating missing values for microarray data, while considering the correlation structure of the data. In order to impute missing samples in the above mentioned motivating CRC case study, the proposed method has been modified to impute expressions for all miRNA of missing normal samples based on multivariate covariates (demographic and lifestyle variables) and to account for the dependence of the imputed data in subsequent differential expression tests. The demographic and lifestyle variables considered in this paper are five continuous (age, number of cigarettes/day, calories, BMI (Body mass index), and lutein and zeaxanthin concentration) and five binary (gender, recent aspirin/NSAID (Non-steroidal anti-inflammatory drug) use, recent smoker, menopause, and post menopause taking HRT (Hormone replacement therapy) within 2 years statuses) variables.

This modified KNN technique imputes all miRNA expression levels of missing normal samples by finding the *k* most similar subjects, not gene expression levels as in conventional KNN-based methods, based on the distance matrices of demographic and lifestyle covariates of patients and produces the variance-covariance matrices for each miRNA. For example, we can estimate the miRNA expression levels in missing normal tissues from a particular subject, based on the expression levels of scanned normal tissues from subjects who have similar demographic and lifestyle covariates.

Another advantage of this method is that it can integrate simultaneously multivariate covariates by aggregating and normalizing their distance matrices (Euclidean, Manhattan, Minkowski, and etc.) to find the nearest neighbor subjects. Specifically, two between-subject distance matrices are constructed based on the fully observed continuous and discrete covariates separately, using Euclidean and Manhattan distances, respectively. These two distance matrices are normalized by scaling between 0 and 1 [18] and aggregated by taking the weighted average of each distance matrix to achieve a single between-subject distance matrix.

### Choice of optimal *k*

There have been many studies carried out to determine the optimal choice (parameter) of *k* for the KNN algorithm. [17] suggest to use the square root of the average number of complete cases after missing data removal, rounded to the nearest odd integer. The simulation studies of different *k* on Likert data [19] show the square root of the number of complete cases which is rounded to the nearest odd integer is a suitable choice for *k*. Moreover, [20] report on *k* = 10 for large data like from microarrays. [8] argue that the imputation method is fairly insensitive to the choice of *k* in the range 10–20. As *k* gets larger, the average distance to the neighbors increases which implies that the imputed value could be less accurate and the imputation time will increase.

However, the choice of a small *k* diminishes the KNN performance because the imputation process overemphasizes a few dominant genes (or subjects in our modification) in estimating the missing values. On the other hand, a large *k* may include genes (or subjects) that are significantly different from the missing values that may result in degrading the imputation performance.

### Accounting for dependence of KNN-imputed data

Because the weighted KNN-imputed expression values are linear combinations of expression values of the fully observed subjects’ expression values, the imputed values are not necessarily independent of the fully observed values. The modified KNN-based imputation method has an advantage of considering this dependence induced by weighted KNN by providing variance-covariance matrices of each miRNA, which can be used when searching for differentially expressed miRNAs. We refer to this method as “KNN dependent”, while referring to the KNN imputation method that ignores the dependence as “KNN independent” in this paper. Its algorithm works almost the same as the algorithms of the conventional KNN-based methods, except it treats the rows as subjects or samples, and the columns as miRNAs.

To see how the proposed imputation method estimates the miRNA expression levels in missing normal samples and accounts for the dependence induced by the weighted KNN, suppose that in the CRC study of *N* subjects, we want to estimate expression levels of *G* miRNAs for normal samples of missing *S* subjects using demographic and lifestyle covariate data. For each normal-missing subject *i*, we find the *k* most similar subjects with non-missing normal samples (say subjects *i*_1_, …, *i*_*k*_), and impute the missing miRNA expression values by multiplying the miRNA expressions from normal samples of the *k* subjects with their corresponding weights, which are generated from the between-subject distance matrix. The imputation of the expression level of miRNA *j* in missing normal sample *i* will be produced as in Eq (2):

$$\begin{array}{c} {\hat{x}}_{ij}=a_{i_{1}}x_{i_{1}j}+a_{i_{2}}x_{i_{2}j}+\ldots +a_{i_{k}}x_{i_{k}j} \end{array}$$ (2)

Here, *i* = 1, …, *S* and *j* = 1, …, *G*. *x*_*lj*_ is the observed expression value of miRNA *j* in the observed normal sample of subject *l*, and *a*_*lj*_ is the weight of the subject in the imputation. The weights *a*_*i*_1__, …, *a*_*i*_*k*__ are obtained as outlined in Eq (1) above. We can generalize Eq (2) to Eq (3):

$$\begin{array}{c} \hat{\underset{∼}{X}}={\underset{∼}{A}}^{T}\underset{∼}{X} \end{array}$$ (3)

Here, $\hat{\underset{∼}{X}}$ is an *S* × *G* matrix of imputed normal tissue expression values, $\underset{∼}{A}$ is a (*N*−*S*) × *S* matrix of weights *a*, and $\underset{∼}{X}$ is a (*N*−*S*) × *G* matrix of observed normal tissue expression values. In column *i* of $\underset{∼}{A}$, the only non-zero elements are in rows *i*_1_, *i*_2_, …, *i*_*k*_, and are the coefficients *a*_*i*_1__, *a*_*i*_2__, …, *a*_*i*_*k*__ in Eq (2).

The variance-covariance matrix of the normal tissue expression for miRNA *j* will be calculated as in Eq (4), assuming the order in the data is the fully observed *N*−*S* subjects followed by the *S* normal-missing subjects:

$$\begin{array}{c} {\underset{∼}{\Sigma }}_{j}\sigma_{j}^{2}=(\begin{array}{cc} \underset{∼}{I} & \underset{∼}{A}\\ {\underset{∼}{A}}^{T} & {\underset{∼}{A}}^{T}\underset{∼}{A} \end{array})\sigma_{j}^{2}, \end{array}$$ (4)

Here, $\sigma_{j}^{2}$ is the variance of miRNA *j* and $\underset{∼}{I}$ is the (*N*−*S*) × (*N*−*S*) identity matrix of non-missing subjects to represent the independence among non-missing subjects. The matrix part of the right-hand side of Eq (4) is denoted by ${\underset{∼}{\Sigma }}_{j}$.

### Testing for differential expression (DE) of miRNA while accounting for dependence

The paired t-test [21] may be used to check whether the miRNAs are differentially expressed in paired normal-tumor samples while accounting for the dependence induced by the imputation method. The paired t-test can be simplified to a one sample t-test of the difference of normal and tumor samples. The per-miRNA null hypothesis is that the difference of mean expression levels of miRNAs between normal and tumor samples is equal to zero. The test statistic for miRNA *j* can be found beginning with the following equation, as discussed in chapter 3 of [22].

$$\begin{array}{c} {\underset{∼}{D}}_{j}=\underset{∼}{1}\mu_{j}+\underset{∼}{\epsilon } \end{array}$$ (5)

Here, ${\underset{∼}{D}}_{j}$ is a *N* × 1 vector of the difference of the *j* ^*th*^ miRNA expressions for normal and tumor samples, *μ*_*j*_ is a single parameter representing the difference of mean expression levels of miRNA *j* between normal and tumor samples, and $\underset{∼}{1}$ is *N* × 1 vector of 1’s. $Var(\underset{∼}{\epsilon })=\sigma_{j}^{2}{\underset{∼}{V}}_{j}$, where ${\underset{∼}{V}}_{j}$ is the variance-covariance matrix of the tumor-normal difference in miRNA expression values for miRNA *j*, i.e., ${\underset{∼}{V}}_{j}=\underset{∼}{I}+{\underset{∼}{\Sigma }}_{j}$, and needs to be a positive definite matrix.

The mean tumor-normal difference for miRNA *j* can be estimated by Eq (6):

$$\begin{array}{c} {\hat{\mu }}_{j}={\left({\underset{∼}{1}}^{T}{{\underset{∼}{V}}_{j}}^{-1}\underset{∼}{1}\right)}^{-1}{\underset{∼}{1}}^{T}{{\underset{∼}{V}}_{j}}^{-1}{\underset{∼}{D}}_{j} \end{array}$$ (6)

The ${\hat{\mu }}_{j}$ in Eq (7) can be substituted from Eq (6):

$$\begin{array}{c} {\hat{\sigma_{j}}}^{2}=\frac{{\left({\underset{∼}{D}}_{j}-\underset{∼}{1}{\hat{\mu }}_{j}\right)}^{-1}{{\underset{∼}{V}}_{j}}^{-1}\left({\underset{∼}{D}}_{j}-\underset{∼}{1}{\hat{\mu }}_{j}\right)}{N-1} \end{array}$$ (7)

Then, the estimated variance of ${\hat{\mu }}_{j}$ would be calculated as in Eq (8):

$$\begin{array}{c} Var\left({\hat{\mu }}_{j}\right)={\hat{\sigma_{j}}}^{2}{\left({\underset{∼}{1}}^{T}{{\underset{∼}{V}}_{j}}^{-1}\underset{∼}{1}\right)}^{-1} \end{array}$$ (8)

Finally, the test statistic will be found using Eq (9) with a degree of freedom of *N*−1.

$$\begin{array}{c} t=\frac{{\hat{\mu }}_{j}}{\sqrt{Var({\hat{\mu }}_{j})}}\cdot \end{array}$$ (9)

This paired t-test can be used with the other imputation methods by replacing ${\underset{∼}{\Sigma }}_{j}$ with the identity matrix, which represents the assumed independence of imputed miRNA values.

### Measuring performance

The performance of the imputation methods on miRNA data is evaluated through root mean squared error (RMSE). The RMSE-based evaluation technique is the most commonly used method to compare similarity between true expression values and imputed expression values. Various variants of RMSE measures are used in the literature: the non-normalized RMSE measure [23] and the normalized RMSE measure by different normalizing constants: average value over all observations in complete data [8], standard deviation of the values in complete data over missing entries [24, 25], and root mean square of the values in complete data over missing entries [26]. However, all above mentioned various RMSE measures provide highly similar results [27].

In the motivating CRC case study, all miRNA expression levels of up to 50% missing normal samples, i.e. up to 50% missing rows (samples) of miRNA data must be imputed. Thus, the non-normalized RMSE that measures the difference between the imputed part of matrix and the original part of matrix, divided by the number of missing cells, can be used. It is calculated as Eq (10):

$$\begin{array}{c} RMSE=\sqrt{\frac{1}{S*G}\sum_{i=1}^{S}\sum_{j=1}^{G}{\left(x_{ij}-{\hat{x}}_{ij}\right)}^{2}} \end{array}$$ (10)

Here, *i* = 1, …, *S* and *j* = 1, …, *G*. *x*_*ij*_ is the original value for missing sample *i* and miRNA *j*, while ${\hat{x}}_{ij}$ is the imputed value for missing sample *i* and miRNA *j*.

## Results

We evaluated the performance of the proposed imputation method, which accounts for the dependence induced by weighted KNN and considers the demographic and lifestyle covariates (KNN dependent), over the weighted KNN ignoring the dependence (KNN independent), MI techniques using MCMC and EM with bootstrapping algorithms, as well as the case deletion technique which only considers fully-observed subjects [9] using simulated data sets.

### Optimal number of nearest neighbor subjects (*k*)

**Fig 1:**
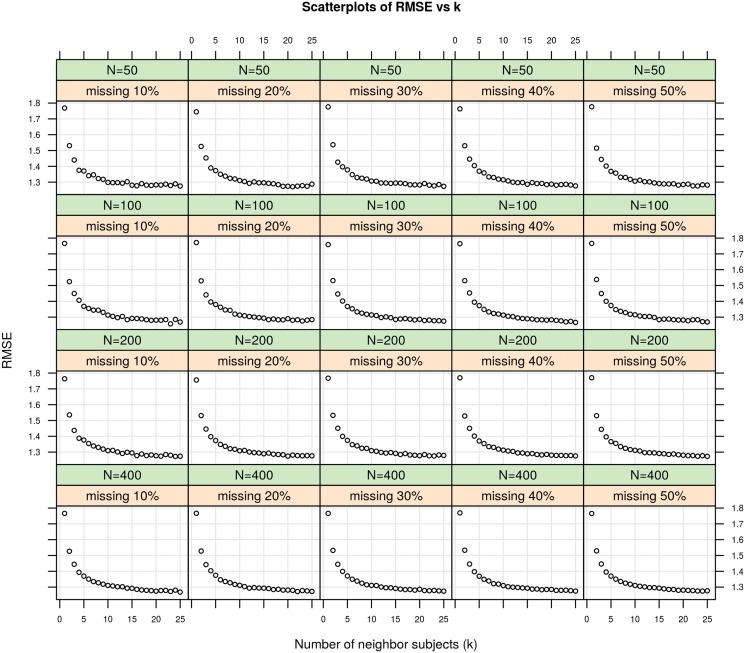
The RMSE values for different number of neighbor subjects (*k*).

Fig 1 shows the effect of the number of neighbor subjects, *k*, used in the KNN imputation method on the RMSE values for simulated data sets with different number of subjects and percent of normal-missing subjects. The RMSE decreases, i.e. the performance of KNN imputation increases, while the value of *k* increases. The falling of RMSE values slows down after *k* value of 10, and becomes approximately the same for the rest of *k* values. The imputation performance becomes approximately insensitive to the value of *k* within the range of 10–25 neighbor subjects. Thus, we used 10 nearest neighbor subjects to estimate the miRNA expression levels of normal samples for missing subjects.

### Simulation data sets

While we have complete normal and tumor sample data for more than 400 subjects in the CRC study, we compare imputation methods using simulated data to have clearly defined power and Type I errors. The imputation analyses were performed on normally distributed paired data matrices of *G* = 2000 miRNA features (columns) for each of the normal and tumor samples with sample sizes of *N* = 50, 100, 200, and 400 subjects (rows). We simulated expression levels of miRNAs for normal and tumor samples by controlling true differentially expressed miRNAs of tumor samples across all simulations. Particularly, all miRNA features of normal samples and only non-differentially expressed miRNA features of tumor samples were simulated based on *μ* = 2 and *σ* = 1.25, while the differentially expressed miRNA features of tumor samples, which consisted of 20% of all miRNA features of tumor samples, were simulated based on *μ* = 2.5 and *σ* = 1.25. This 20% differential expression rate as well as this mean tumor-normal difference of 2.5 and standard deviation of 1.25 were chosen based on characteristics of the motivating CRC study. We randomly applied missingness from 10 to 50 percent of the normal data rows. We performed 25 simulations for each sample size with different percent missingness.

To ensure that the simulated data sets reflected the characteristics of the CRC study, and that the demographic and lifestyle variables carried some useful information for imputation, the multivariate covariate data sets with demographic and lifestyle variables of subjects were simulated based on *z* randomly selected true differentially expressed miRNA expression levels using the characteristics of the CRC case study covariate data. For example, a continuous variable such as age of subjects was simulated as in Eq (11):

$$\begin{array}{c} \hat{C}=\beta_{0}+\sum_{j=1}^{z}\beta_{j}x_{j}+\epsilon \end{array}$$ (11)

Here, *j* = 1, …, *z*, $\hat{C}$ is a simulated value of age, *β*_0_ is the mean age of the patients in CRC case study, and *β*_*j*_ is uniformly distributed with a minimum and a maximum of up to 5% of the minimum and the maximum of the CRC case study patients’ age, respectively. In this paper, we used 2% of the minimum and the maximum of the continuous variables with *z* = 20, which was selected for computational simplicity, to simulate variables with similar characteristics of CRC case study covariates. *x*_*j*_ is the expression of truly differentially expressed miRNA *j* in tumor, and the error term *ϵ* is normally distributed with zero mean (*μ* = 0) and variance of 10% of variance of the patients’ age ($\sigma^{2}=0.1*\sigma_{age}^{2}$).

The binary variables such as gender of subjects was simulated using a logistic regression model in Eqs (12) and (13):

$$\begin{array}{c} log\frac{p}{1-p}=\beta_{0}+\sum_{j=1}^{z}\beta_{j}x_{j} \end{array}$$ (12)

Here, *p* is the probability of *gender = female*, say.

Eq (12) can be rewritten as Eq (13):

$$\begin{array}{c} \hat{P}={\left[1+exp\left(-\left(\beta_{0}+\sum_{j=1}^{z}\beta_{j}x_{j}\right)\right)\right]}^{-1} \end{array}$$ (13)

Here, $\hat{P}$ is a simulated probability of *gender = female*, *β*_0_ is the mode of the patients’ gender in the CRC case study, and *β*_*j*_ is uniformly distributed as *U*[−0.5, 0.5]. To ensure variability in simulated binary variables, we calculate ${\hat{P}}^{\prime }$ as in Eq (14):

$$\begin{array}{c} {\hat{P}}^{'}=\frac{\hat{P}-min\left(\hat{P}\right)}{max\left(\hat{P}\right)-min\left(\hat{P}\right)}\cdot \end{array}$$ (14)

In our simulated study, we had denoted as a male if the value of ${\hat{P}}^{\prime }$ was between 0 and 0.5, and as a female if the ${\hat{P}}^{\prime }$ was bigger than 0.5 but less than or equal to 1.

Demographic and lifestyle variables were thus simulated based on characteristics of five continuous (age, number of cigarettes/day, calories, BMI, and lutein and zeaxanthin concentration) and five binary (gender, recent aspirin/NSAID use, recent smoker, menopause, and post menopause taking HRT within 2 years statuses) variables from the CRC study.

We carried out the performance analyses as follows: First, we called arbitrarily the subjects with missing normal samples. Then, we imputed expression levels of the missing normal samples using the imputation methods mentioned in the Methods section. We evaluated the performance of these imputation methods against the initial generated data matrices by calculating the RMSE for such simulated data set. Moreover, we carried out the differential expression (DE) analyses on the imputed data sets to check whether the KNN dependent method has an equal statistical power in finding differentially expressed miRNA as other imputation techniques.

### Performance of imputation techniques

**Fig 2:**
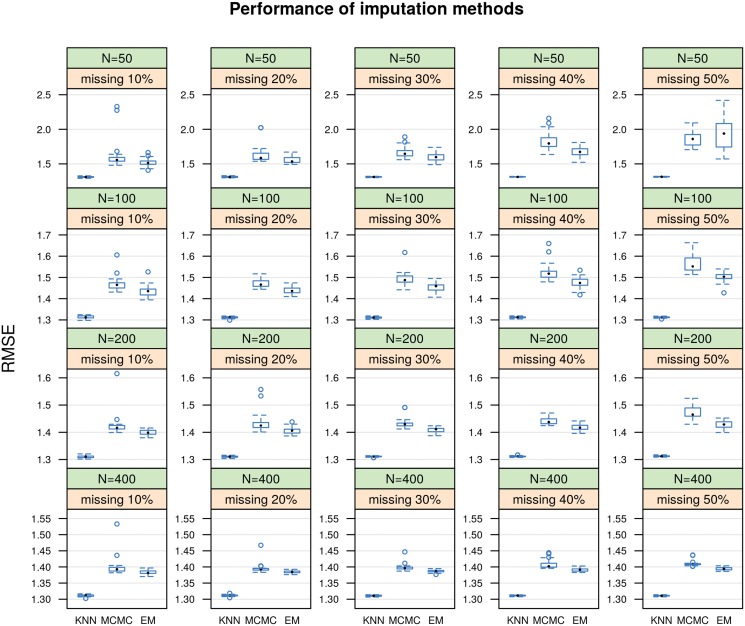
The RMSE values for different imputation techniques.

The performance of the modified KNN method was assessed over MI techniques using MCMC and EM with bootstrapping algorithms for data matrices with different number of subjects and different percents of normal missing subjects. In Fig 2, the modified KNN method shows consistently better performance than other imputation techniques (systematically lower RMSE values) for sample sizes of 50, 100, 200, and 400 subjects, with missing percentages of 10–50.

The KNN imputation method also shows a robustness to increasing the percent of missing normal samples and the number of subjects in miRNA data sets. It keeps relatively the same performance for all levels of missing percents and number of subjects.

Moreover, the KNN imputation method required much less computational expense than the MI techniques using MCMC and EM with bootstrapping algorithms. For example, to impute the expressions of 50% missing normal samples in 400 subjects on a machine with CPU speed of 1.86 GHz and 2 GB RAM, the KNN method took approximately 35 minutes, whereas MCMC and EM with bootstrapping algorithms took approximately 10 and 5 hours, respectively.

### Differential expression (DE) testing

We applied the paired t-test to the data sets, which were imputed by various imputation methods, to see how well we could identify differentially expressed miRNAs. First, we obtained a test statistic and a p-value for each miRNA feature in each imputed data set by controlling the false discovery rate (FDR) at 0.05 within each simulation. Then, we calculated the true positive rate (TPR), the false positive rate (FPR), and the false discovery rate (FDR) based on the miRNAs which were controlled as truly differentially expressed in the simulations. The TPR and FPR were defined and calculated as in [28] and [29], and the FDR was defined as in [30].

**Fig 3:**
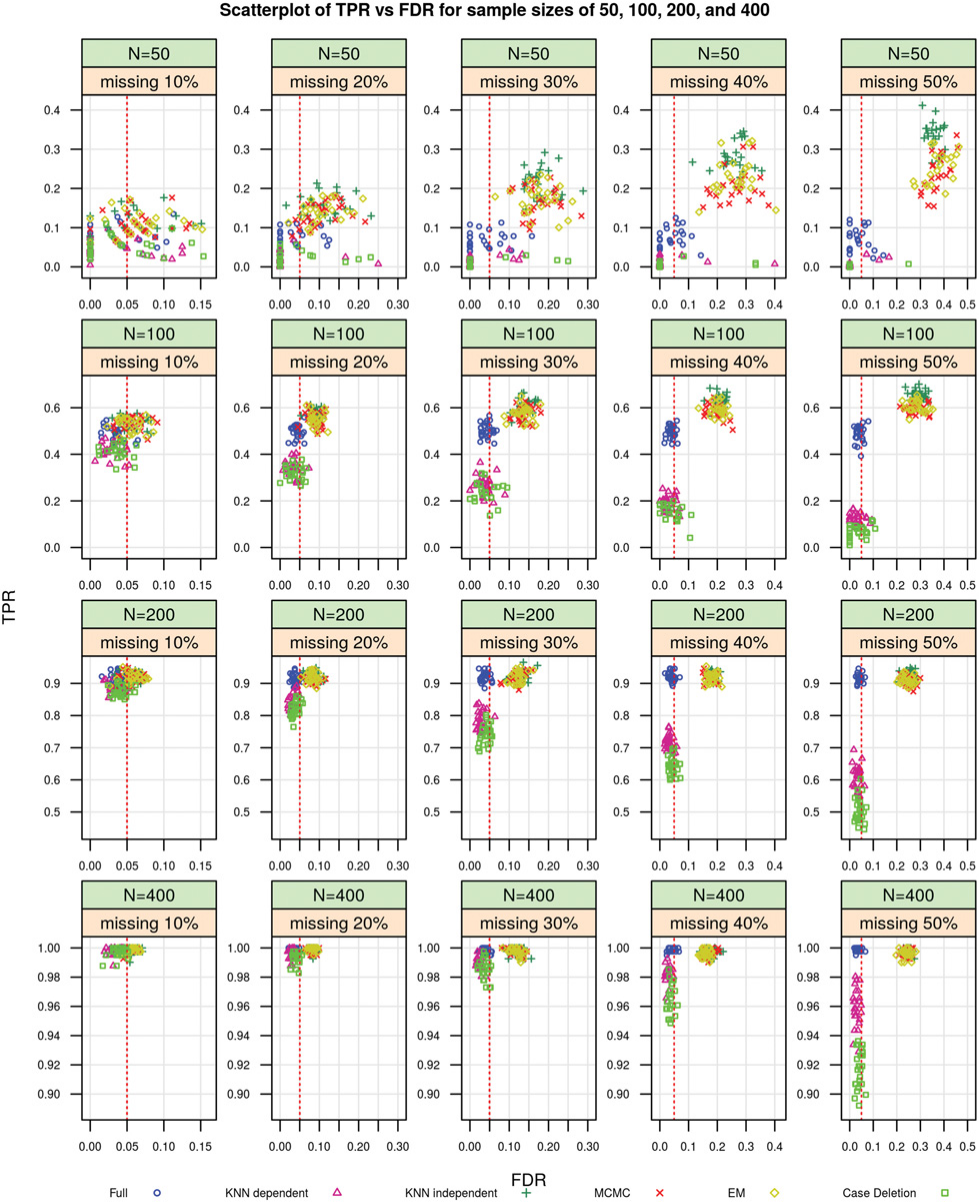
TPR and FDR for sample sizes of 50, 100, 200, and 400 with missingness of 10%–50%.

Fig 3 shows the performance (including power and FDR control) of the paired t-tests on the data sets imputed by the KNN dependent, the KNN independent, the MI using MCMC and MI using EM algorithms, the case deletion technique, as well as on the full data set for the number of subjects of 50, 100, 200, and 400 with the missing percent of normal samples of 10%–50%. The scatter plots of TPR and FPR for the similar conditions are represented in S1 Fig (see Supplemental materials).

From Fig 3 we can see that the power (i.e., the TPR values) increases with larger sample sizes. For 400 subjects and 50% missing normal samples, which are the characteristics of the CRC case study, there are clear clusterings of TPR and FDR values, separately for full, for KNN dependent and case deletion, and for KNN independent, MCMC, and EM methods. Although the KNN dependent has slightly lower power than the other imputation methods (the TPR values are in the range of 0.93–0.98 for 400 subjects and 50% missing), it controls the FDR values below the threshold of 0.05, which is represented by red dotted lines in the figures. The KNN independent, the MCMC, and the EM with bootstrapping algorithms have the highest power (the TPR values are in the range of 0.985-1 for 400 subjects and 50% missing), but lack control of the FDR, i.e. the FDR values cross the threshold of 0.05 for all number of subjects and missing percentages. The case deletion method shows the lowest power, but maintains control of the FDR for all number of subjects and percentages of missing normal samples.

## Discussion

The imputation accuracy of the proposed KNN imputation method, using the aggregated metric distance matrices of the demographic and lifestyle data, in the simulation data sets was higher than that of the MI methods using MCMC and EM with bootstrapping algorithms. Moreover, the proposed KNN method was robust and imputed the miRNA features of missing normal samples with less computational expense than the other imputation methods.

The DE tests of the KNN imputed data sets show that the KNN method while accounting for the dependence of the imputed values (KNN dependent) provided greater power than if no imputation were done (the case deletion approach) and maintained control of the FDR. The KNN method while ignoring the dependence (KNN independent), as well as MCMC and EM with bootstrapping algorithms had higher power than the power of KNN dependent, but failed to control the FDR. These effects are more clear for larger missing percents and number of subjects.

Depending on the study goals, researchers could select the KNN method while ignoring the dependence (achieving more power and higher proportion of false discoveries) or considering the dependence (moderate loss of power but lower proportion of false discoveries). In the motivating CRC study, the chosen approach is the KNN method while accounting for the dependence, with moderate loss of power but maintaining control of the FDR.

The case deletion method showed the lowest power to identify differentially expressed miRNAs, though it had similar FDR control as the KNN dependent method.

In this paper, we applied the paired t-test to identify differentially expressed miRNAs from normally distributed simulated miRNA data while accounting for the dependence structure of the imputed data. However, miRNA data can be noisy and not normally distributed. Currently available nonparametric tests may also not be directly applicable because they assume independence. In this respect, it is challenging to construct a statistical model which tests for significant miRNAs from paired samples while accounting for the dependence. Our future work is to develop a nonparametric t-test method which enables paired t-tests on a large number of miRNA data, using permutations with manageable computational expense, while accounting for the dependence induced by KNN imputation.

## Supplemental materials

S1 Fig shows the scatter plots of TPR and FPR of the KNN dependent and independent methods, the MI techniques using MCMC and EM with bootstrapping algorithms, as well as full and case deletion techniques. The R code to generate the simulated data are also provided (in a.zip file) as S1 File.

## Supporting Information

S1 Fig TPR and FPR for sample sizes of 50, 100, 200, and 400 with missingness of 10%–50%. (TIF)

S1 File R code to generate the simulated data, beginning with README.txt file. (ZIP)

## References

[1] Lee RC, Feinbaum RL, Ambros V (1993) The C. elegans heterochronic gene lin-4 encodes small RNAs with antisense complementarity to lin-14. Cell 75: 843–854. 10.1016/0092-8674(93)90529-Y 8252621

[2] Esquela-Kerscher A, Slack FJ (2006) Oncomirs-microRNAs with a role in cancer. Nature Reviews Cancer 6: 259–269. 10.1038/nrc1840 16557279

[3] Calin GA, Croce CM (2006) MicroRNA signatures in human cancers. Nature Reviews Cancer 6: 857–866. 10.1038/nrc1997 17060945

[4] Michael MZ, O’Connor SM, van Holst Pellekaan NG, Young GP, James RJ (2003) Reduced accumulation of specific microRNAs in colorectal neoplasia. Molecular Cancer Research 1: 882–891. 14573789

[5] Yang L, Belaguli N, Berger DH (2009) MicroRNA and colorectal cancer. World Journal of Surgery 33: 638–646. 10.1007/s00268-008-9865-5 19123024

[6] Akao Y, Nakagawa Y, Naoe T (2006) let-7 microRNA functions as a potential growth suppressor in human colon cancer cells. Biological and Pharmaceutical Bulletin 29: 903–906. 10.1248/bpb.29.903 16651716

[7] He L, He X, Lim LP, De Stanchina E, Xuan Z, Liang Y, et al (2007) A microRNA component of the p53 tumour suppressor network. Nature 447: 1130–1134. 10.1038/nature05939 17554337 PMC4590999

[8] Troyanskaya O, Cantor M, Sherlock G, Brown P, Hastie T, Tibshirani R, et al (2001) Missing value estimation methods for DNA microarrays. Bioinformatics 17: 520–525. 10.1093/bioinformatics/17.6.520 11395428

[9] Little RJ, Rubin DB (2002) Statistical analysis with missing data.

[10] Dempster AP, Laird NM, Rubin DB (1977) Maximum likelihood from incomplete data via the EM algorithm. Journal of the Royal Statistical Society Series B (Methodological): 1–38.

[11] Honaker J, King G (2010) What to do about missing values in time-series cross-section data. American Journal of Political Science 54: 561–581. 10.1111/j.1540-5907.2010.00447.x

[12] Rubin DB (2004) Multiple imputation for nonresponse in surveys 81.

[13] Kim KY, Kim BJ, Yi GS (2004) Reuse of imputed data in microarray analysis increases imputation efficiency. BMC Bioinformatics 5: 160 10.1186/1471-2105-5-160 15504240 PMC528735

[14] Schafer JL (2010) Analysis of incomplete multivariate data. CRC press.

[15] Yuan YC (2010) Multiple imputation for missing data: Concepts and new development (version 9.0). SAS Institute Inc, Rockville, MD.

[16] Cover T, Hart P (1967) Nearest neighbor pattern classification. Information Theory, IEEE Transactions on 13: 21–27. 10.1109/TIT.1967.1053964

[17] Duda PE, Richard O (1973) Hart, pattern classification and scene analysis.

[18] Visalakshi NK, Thangavel K (2009) Impact of normalization in distributed k-means clustering. International Journal of Soft computing 4: 168–172.

[19] Jonsson P, Wohlin C (2004) An evaluation of k-nearest neighbour imputation using likert data. Software Metrics, 2004 Proceedings 10th International Symposium on: 108–118.

[20] Batista G, Monard MC (2001) A study of K-nearest neighbour as a model-based method to treat missing data. Argentine Symposium on Artificial Intelligence.

[21] Student (1908) The probable error of a mean. Biometrika: 1–25.

[22] Seber GA, Lee AJ (2003) Linear regression analysis. John Wiley & Sons.

[23] Bø TH, Dysvik B, Jonassen I (2004) LSimpute: accurate estimation of missing values in microarray data with least squares methods. Nucleic acids research 32: e34–e34. 10.1093/nar/gnh026 14978222 PMC374359

[24] Oba S, Sato Ma, Takemasa I, Monden M, Matsubara Ki, Ishii S (2003) A Bayesian missing value estimation method for gene expression profile data. Bioinformatics 19: 2088–2096. 10.1093/bioinformatics/btg287 14594714

[25] Kim H, Golub GH, Park H (2005) Missing value estimation for DNA microarray gene expression data: local least squares imputation. Bioinformatics 21: 187–198. 10.1093/bioinformatics/bth499 15333461

[26] Ouyang M, Welsh WJ, Georgopoulos P (2004) Gaussian mixture clustering and imputation of microarray data. Bioinformatics 20: 917–923. 10.1093/bioinformatics/bth007 14751970

[27] Oh S, Kang DD, Brock GN, Tseng GC (2011) Biological impact of missing-value imputation on downstream analyses of gene expression profiles. Bioinformatics 27: 78–86. 10.1093/bioinformatics/btq613 21045072 PMC3008641

[28] Bolstad BM (2004) Low-level analysis of high-density oligonucleotide array data: background, normalization and summarization Ph.D. thesis, University of California, Berkeley.

[29] Stevens JR, Bell JL, Aston KI, White KL (2010) A comparison of probe-level and probeset models for small-sample gene expression data. BMC Bioinformatics 11: 281 10.1186/1471-2105-11-281 20504334 PMC2901368

[30] Benjamini Y, Hochberg Y (1995) Controlling the false discovery rate: a practical and powerful approach to multiple testing. Journal of the Royal Statistical Society Series B (Methodological): 289–300.

