# Supplementary figures and images for: Accounting for Dependence Induced by Weighted KNN Imputation in Paired Samples, Motivated by a Colorectal Cancer Study
Source: PLoS One. 2015 Apr 7;10(4):e0119876. doi: 10.1371/journal.pone.0119876 (PMC4388652; doi:10.1371/journal.pone.0119876)

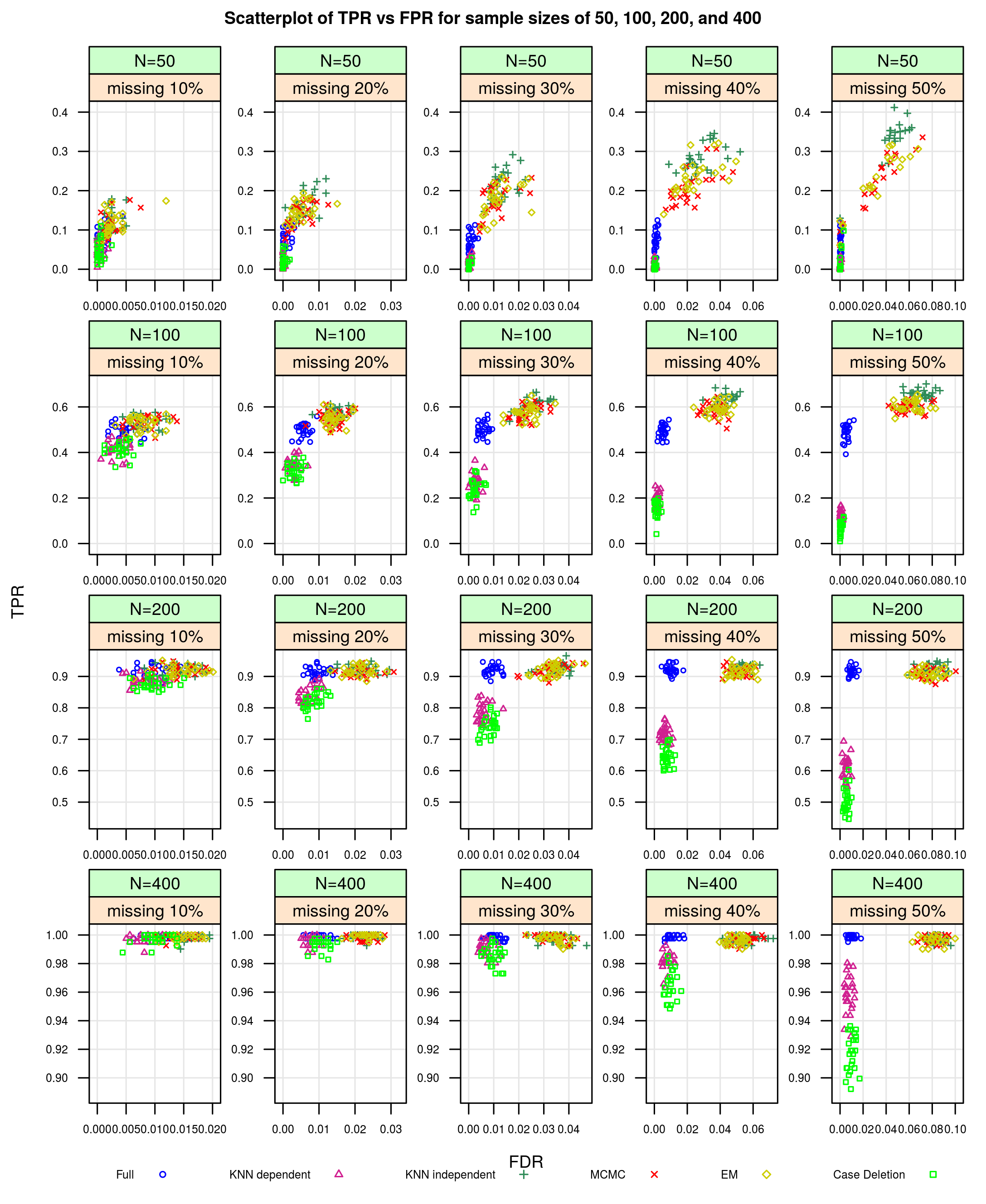

Supplement: S1 Fig — (TIF) [file pone.0119876.s001.tif]
